# Expression Profiling of Stem Cell-Related Genes in Neoadjuvant-Treated Gastric Cancer: A *NOTCH2*, *GSK3B* and *β-catenin* Gene Signature Predicts Survival

**DOI:** 10.1371/journal.pone.0044566

**Published:** 2012-09-10

**Authors:** Lukas Bauer, Rupert Langer, Karen Becker, Alexander Hapfelmeier, Katja Ott, Alexander Novotny, Heinz Höfler, Gisela Keller

**Affiliations:** 1 Institute of Pathology, Technische Universität München, München, Germany; 2 Department of Surgery, Universität Heidelberg, Heidelberg, Germany; 3 Department of Surgery, Technische Universität München, München, Germany; 4 Institute of Medical Statistics and Epidemiology, Technische Universität München, München, Germany; 5 Institute of Pathology, Helmholtz-Zentrum München, Neuherberg, Germany; University of Texas MD Anderson Cancer Center, United States of America

## Abstract

Cancer stem cell (CSC) based gene expression signatures are associated with prognosis in various tumour types and CSCs are suggested to be particularly drug resistant. The aim of our study was first, to determine the prognostic significance of CSC-related gene expression in residual tumour cells of neoadjuvant-treated gastric cancer (GC) patients. Second, we wished to examine, whether expression alterations between pre- and post-therapeutic tumour samples exist, consistent with an enrichment of drug resistant tumour cells. The expression of 44 genes was analysed in 63 formalin-fixed, paraffin embedded tumour specimens with partial tumour regression (10–50% residual tumour) after neoadjuvant chemotherapy by quantitative real time PCR low-density arrays. A signature of combined *GSK3B* ^high^, *β-catenin (CTNNB1)* ^high^ and *NOTCH2* ^low^ expression was strongly correlated with better patient survival (p<0.001). A prognostic relevance of these genes was also found analysing publically available gene expression data. The expression of 9 genes was compared between pre-therapeutic biopsies and post-therapeutic resected specimens. A significant post-therapeutic increase in *NOTCH*2, *LGR5* and *POU5F1* expression was found in tumours with different tumour regression grades. No significant alterations were observed for *GSK3B* and *CTNNB1*. Immunohistochemical analysis demonstrated a chemotherapy-associated increase in the intensity of NOTCH2 staining, but not in the percentage of NOTCH2. Taken together, the *GSK3B, CTNNB1* and *NOTCH2* expression signature is a novel, promising prognostic parameter for GC. The results of the differential expression analysis indicate a prominent role for NOTCH2 and chemotherapy resistance in GC, which seems to be related to an effect of the drugs on NOTCH2 expression rather than to an enrichment of NOTCH2 expressing tumour cells.

## Introduction

Advanced gastric carcinomas (GC) are frequently treated by platin/5-fluorouracil (5FU)- based neoadjuvant chemotherapy [1]. The aim of this therapy is, amongst others, to shrink the tumour before surgery to increase the probability of complete resection and to thus improve patient survival. However, response rates are low, and complete or subtotal tumour regression is observed in only 20–40% of the patients [1], [2]. Thus, chemotherapy resistance is a major obstacle for successful treatment.

According to the cancer stem cell hypothesis, tumour cells are heterogeneous, and an increased drug resistance is a particular phenotype of a minority of tumour cells – the so-called cancer-initiating cells or cancer stem cells (CSCs) [3]–[5]. An increase in the CSC population after chemotherapy has been demonstrated [6], [7], and stem cell based gene expression signatures were associated with poor prognosis in various tumours including gastric carcinomas [8]–[11]. The CSC hypothesis is still controversially discussed, but there is evidence for the existence of CSCs in several tumour types and molecular markers have been identified which are preferentially found on these cells [4], [5]. The activation of embryonic signalling pathways, such as the Wnt, Notch and Hedgehog pathways, has been suggested as a driving force for the formation of CSCs [4], [12]. Data regarding the source and existence of gastric CSCs remain inconclusive [13]–[17]. In mice, bone-marrow derived cells or a specific cell population in the antrum expressing the Wnt target molecule LGR5, have been associated with CSCs in the stomach [17], [18]. In addition, CD44 and CD24 have been suggested as specific cell surface markers, but the data are inconsistent [19], [20].

The neoadjuvant treatment protocol for GC provides an excellent opportunity to investigate tumour cells before and after chemotherapy in patients. In this study, we aimed to elucidate first, whether the expression of putative CSC-related genes in the post-therapeutic residual tumour predicts patient survival and second, whether particular genes are differentially expressed between pre-therapeutic biopsies and the post-therapeutic tumour specimens, consistent with an enrichment of chemotherapy-resistant tumour cells as predicted by the CSC concept. The group of patients, who demonstrated considerable tumour shrinkage after neoadjuvant chemotherapy, but still had sufficient residual tumour cells available for analysis (10–50% residual tumour) were considered as the most suitable group to start with a screening analysis for prognostic relevant genes and to then identify relevant differences in gene expression between the pre- and post-therapeutic tumour samples. Analysing these residual tumour cells we identified a gene expression pattern encompassing *GSK3B, CTNNB1* and *NOTCH2*, which strongly predicts prognosis of the patients. We show that the impact of *GSK3B* and *CTNNB1* to this signature is not dependent on chemotherapy and more likely reflects a property of the primary tumour and our data further suggest, that in particular *NOTCH2* might play a role for chemotherapy resistance in GC.

## Materials and Methods

### Patients

In total, 480 patients with locally advanced GC (cT3/4) were treated by neoadjuvant, platin/5FU-based chemotherapy at the Department of Surgery at the Technische Universität München between 1991 and 2007 and were evaluated for response based on a standardized histopathological protocol [2]–[22]. Tumour regression was classified into 3 grades: tumour regression grade (TRG) 1, which consists of TRG1a (total tumour regression) and TRG1b (subtotal tumour regression: <10% residual tumour cells/tumour bed), TRG2 (partial tumour regression: 10–50% residual tumour cells/tumour bed) and TRG3 (minimal or no tumour regression: >50% residual tumour/tumour bed). Of the 480 patients, 121 patients demonstrated TRG2 and 63 of these were analysed in this study. The inclusion criterion was the availability of sufficient tumour tissue for the analysis of patients treated with at least 50% of the projected dose of chemotherapy. Patient characteristics and treatment protocols are shown in Table 1. To confirm the representative nature of the 63 analysed patients, the distribution of their clinicopathological parameters was compared to the 121-patient cohort and revealed no statistically significant differences.

**Table 1:** Patient characteristics and treatment.

Variable	Category		n (%)
Patients			63 (100)
Age [yrs]	median	57.6	
	range	35.0 – 73.0	
Sex	female		16 (25)
	male		47 (74)
Tumour localisation	proximal		43 (68)
	medial		12 (19)
	distal		7 (11)
	total		1 (1)
Lauren classification	intestinal		24 (38)
	non-intestinal		39 (61)
Tumour grade	G1+2		5 (7)
	G3		58 (92)
Neoadjuvant chemotherapy	PLF^1^		48 (76)
	OLF^2^		3 (4)
	Epirubicin-PLF^3^		4 (6)
	Paclitaxel/Docetaxel-PLF^4^		8 (12)
Resection category	R0		53 (84)
	R1		10 (15)
ypT category^5^	ypT0-2		46 (73)
	ypT3+4		17 (27)
ypN category^5^	ypN0		24 (38)
	ypN1-3		39 (61)
ypM category^5^	ypM0		52 (82)
	ypM1		11 (17)

1 Preoperative chemotherapy protocol: PLF: two cycles, each consisting of cisplatin (50 mg/m^2^ body surface area (BSA)) at weeks 1, 3 and 5, and both leucovorin (500 mg/m^2^ BSA) and 5-fluorouracil (2000 mg/m^2^ BSA) at weeks 1, 2, 3, 4, 5 and 6 (PLF),

2 Oxaliplatin (85 mg/m^2^ BSA) replaces cisplatin in PLF.

3 Additional epirubicin (30 mg/m^2^ BSA) at weeks 2, 4, and 6,

4 Additonal paclitaxel (85 mg/m^2^ BSA) or docetaxel (40–50 mg/m^2^ BSA) at weeks 1, 3, and 5,

5 TNM Classification of Malignant Tumors, 6^th^ Edition, UICC.

Follow-up was calculated from the first day of treatment until the date of last contact with the patients. The median follow-up was 77.1 months (range: 28.5–108.5). The clinical endpoint of the study was overall survival (OS), which was defined as the time between the first day of chemotherapy and death by any cause. The median OS was 50.9 months (range: 4.5–108.5, 95% CI: 25.6–76.3), and 37 of the 63 patients died during follow up. This sample size and number of events are sufficiently large for a consistent estimation of all effect sizes investigated in this explorative study [23], [24].

The comparison of gene expression between corresponding pre- and post-therapeutic tumour samples was performed for patients with TRG2 and TRG3 (each n = 22).

For the immunohistochemical analysis, pre- and post-therapeutic tumour samples from 21 patients with TRG1b, 21 patients with TRG2, 22 patients with TRG3 and of 16 patients treated by surgery alone were included. The selection criterion for these analyses was the availability of corresponding pre-therapeutic biopsies and post-therapeutic tumours.

### Ethics Statement

The study and the use of human tissues was approved by the local Institutional Review Board at the Technische Universität München (reference: 2158/08), and informed consent was obtained according to institutional regulations.

### RNA Extraction and Reverse Transcription

Total RNA was extracted from formalin-fixed, paraffin-embedded (FFPE) tissues after manual microdissection of tumour areas composed of at least 50% tumour cells. The RNA was purified by phenol and chloroform extraction and was reverse transcribed as described [25].

### Gene Expression Analysis

The 44 genes that were selected for analysis based on their potential role in CSC biology are included in Table 2. Gene expression was analysed by quantitative real time PCR (qRT-PCR) on custom-made TaqMan® low density arrays (Applied Biosystems Inc., Foster City, USA). Appropriate reference genes were determined by an analysis of ten candidate reference genes in 8 gastric carcinomas using the geNorm-algorithm [26]. *IPO8*, *POLR2A* and *UBC* were determined to be the most suitable reference genes and normalisation based on the geometric mean of these three genes was performed as described [26]. Reagents, cycling conditions and software are included in the Supporting Information. Relative gene expression was quantified using the comparative ΔΔCt method [27].

**Table 2:** Genes analysed by TaqMan® low density array.

Gene	Gene ID^1^	AssayID^2^	Amplicon-length [bp]
*ABCB1*	5243	Hs00184500_m1	67
*ABCG2*	9429	Hs01053790_m1	83
*ALDH1A1*	216	Hs00946916_m1	61
*ASCL2*	430	Hs00270888_s1	101
*ATXN1*	6310	Hs00165656_m1	97
*AXIN1*	8312	Hs00394718_m1	81
*BMI1*	648	Hs00180411_m1	105
*CCND1*	595	Hs00765553_m1	57
*CD133*	8842	Hs01009257_m1	80
*CD24*	100133941	Hs02379687_s1	140
*CD34*	947	Hs02576480_m1	63
*CD44*	960	Hs01075861_m1	70
*CDH1*	999	Hs01013953_m1	65
*CDX2*	1045	Hs01078080_m1	81
*CHD1*	1105	Hs00154405_m1	84
*CTNNB1*	1499	Hs00355045_m1	86
*DKK3*	27122	Hs00247426_m1	83
*DNMT1*	1786	Hs00154749_m1	77
*DNMT3A*	1788	Hs01027166_m1	79
*DNMT3B*	1789	Hs00171876_m1	55
*FOXD3*	27022	Hs00255287_s1	73
*FZD1*	8321	Hs00268943_s1	83
*GADD45A*	1647	Hs00169255_m1	123
*GLI1*	2735	Hs00171790_m1	80
*GSK3B*	2932	Hs00275656_m1	73
*HDAC1*	3065	Hs00606262_g1	149
*HDAC2*	3066	Hs00231032_m1	106
*IHH*	3549	Hs01081801_m1	103
*KLF4*	9314	Hs00358836_m1	110
*LGR4*	55366	Hs00173908_m1	68
*LGR5*	8549	Hs00173664_m1	112
*LIN28*	79727	Hs00702808_s1	143
*MKI67*	4288	Hs01032443_m1	66
*MYC*	4609	Hs00905030_m1	87
*NANOG*	79923	Hs02387400_g1	109
*NOTCH1*	4851	Hs01062014_m1	80
*NOTCH2*	4853	Hs01050719_m1	60
*OLFM4*	10562	Hs00197437_m1	85
*POU5F1*	5460	Hs00999632_g1	77
*PTCH1*	5727	Hs00970979_m1	63
*SFRP1*	6422	Hs00610060_m1	130
*SHH*	6469	Hs00179843_m1	70
*SMO*	6608	Hs01090242_m1	54
*SOX2*	6657	Hs01053049_s1	91
*IPO8* 3	10526	Hs00183533_m1	71
*POLR2A* 3	5430	Hs00172187_m1	61
*UBC* 3	7316	Hs00824723_m1	71

1 www.ncbi.nlm.nih.gov/gene,

2 Applied Biosystems,

3 Reference genes.

### Immunohistochemistry

The monoclonal NOTCH2 antibody (C651.6DbHN) was obtained from the Developmental Studies Hybridoma Bank (DSHB, The University of Iowa, Department of Biology, Iowa City, USA). The staining procedure and examination of antibody specificity by Western blotting are described in the Supporting Information and Figure S1A.

Immunohistochemical staining was scored in a blinded fashion by two independent researchers (L.B. and R.L). Cytoplasmic and nuclear staining was evaluated separately. Negative, weak, medium or strong staining intensities were scored as 0, 1, 2 and 3, respectively. The percentage of tumour cells with stained cytoplasm/nucleus was scored as 0 (negative), 1 (<10%), 2 (10 to <50%), 3 (50 to <80%) and 4 (≥80%).

### Statistical Analysis

Conditional inference tests were used to determine the optimal cut-off-values of gene expression for the association with patient survival and to determine the p-values appropriate for maximally selected statistics [28]. Gene expression values above or equal to the optimal cut-off value were defined as high expression and gene expression values below the cut-off value were defined as low expression. Survival rates were estimated according to Kaplan-Meier curves and were compared by log-rank tests. Relative risks were estimated by determining hazard ratios (HRs) from Cox proportional hazard models. In the multivariate analysis, stepwise forward variable selection was performed based on likelihood ratio tests. The ratio of the number of variables in the model to the number of events was limited to 1∶10 [24].

Comparisons of clinicopathological variables between groups were performed by Mann-Whitney-U tests. The χ^2^-test and Fisher’s exact test were used for the comparison of relative frequencies where appropriate. Statistical differences of gene expressions between paired samples were analysed using the Wilcoxon signed rank test.

Unsupervised hierarchical cluster analysis was performed with Cluster 3.0 software [29] and Java TreeView software (version 1.1.5r2) [30]. Relative mRNA expression data were log-transformed, median-centred and normalised before applying complete linkage clustering with a distance matrix based on Pearson’s correlation (uncentred).

A risk score was calculated by summarizing the products of the multiplication of the Cox regression coefficient of each gene in the model with the normalised gene expression values for each patient essentially as described [31] (details in Material and Methods S1 and Tables S4 and S6).

All statistical tests were two-sided and conducted in an explorative manner with a significance level of 0.05 using the SPSS 18.0 software (SPSS Inc., Chicago, IL 11.5) and R (R Foundation for Statistical Computing, Vienna, Austria).

The study complies with the reporting recommendations for tumour marker prognostic studies (REMARK criteria) [32].

### Analysis of Public Microarray Data

Publically available gene expression array data of gastric carcinomas with descriptions of clinical characteristics and patient survival [33] were obtained from the BRB-Array Tools data archive (http://linus.nci.nih.gov/~brb/DataArchive_New.html) and analysed using BRB-Array Tools [34] (Supporting Information). Only advanced gastric carcinomas (T3/4) (n = 58) were included in the analysis. The optimal cut-off values of *GSK3B*, *CTNNB1*, and *NOTCH2* expression were determined for an association with patient survival by the conditional inference tests for maximally selected statistics as described above.

## Results

### Gene Expression and Patient Survival

The gene expression profiling of the 63 tumours of patients with TRG2 showed, that high expression levels of *GSK3B*, *DNMT1* and *CTNNB1* were significantly associated with better survival (conditional inference test: p = 0.006, 0.041, and 0.043, respectively). A moderate association with better survival was observed for high expression of *ABCG2* and *OLFM4* (p = 0.051, p = 0.055) and for low expression of *NOTCH2* (p = 0.071) (Table 3). Univariate Cox-regression analysis demonstrated approximately concordant results (Table 4).

**Table 3:** Gene expression and association with survival – conditional inference tests.

	< cut-off		≥ cut-off		
Gene	n	median survival [mo]	n	median survival [mo]	p-value
*GSK3B*	40	47.1	23	102.6	0.006
*DNMT1*	53	42.1	10	nr	0.041
*CTNNB1*	15	32.3	48	94.9	0.043
*ABCG2*	54	47.1	9	102.6	0.051
*OLFM4*	53	40.4	10	nr	0.055
*NOTCH2*	31	94.9	32	40.4	0.071

nr: median survival not reached.

A multivariate Cox regression analysis including *GSK3B*, *CTNNB1*, *DNMT1* and the standard prognostic variables in GC, namely ypT, ypN, ypM and resection category revealed *GSK3B* as the second most important independent prognostic factor (HR: 0.128, 95% CI: 0.033–0.492, p = 0.003) after distant metastasis (Table S1).

**Figure 1:**
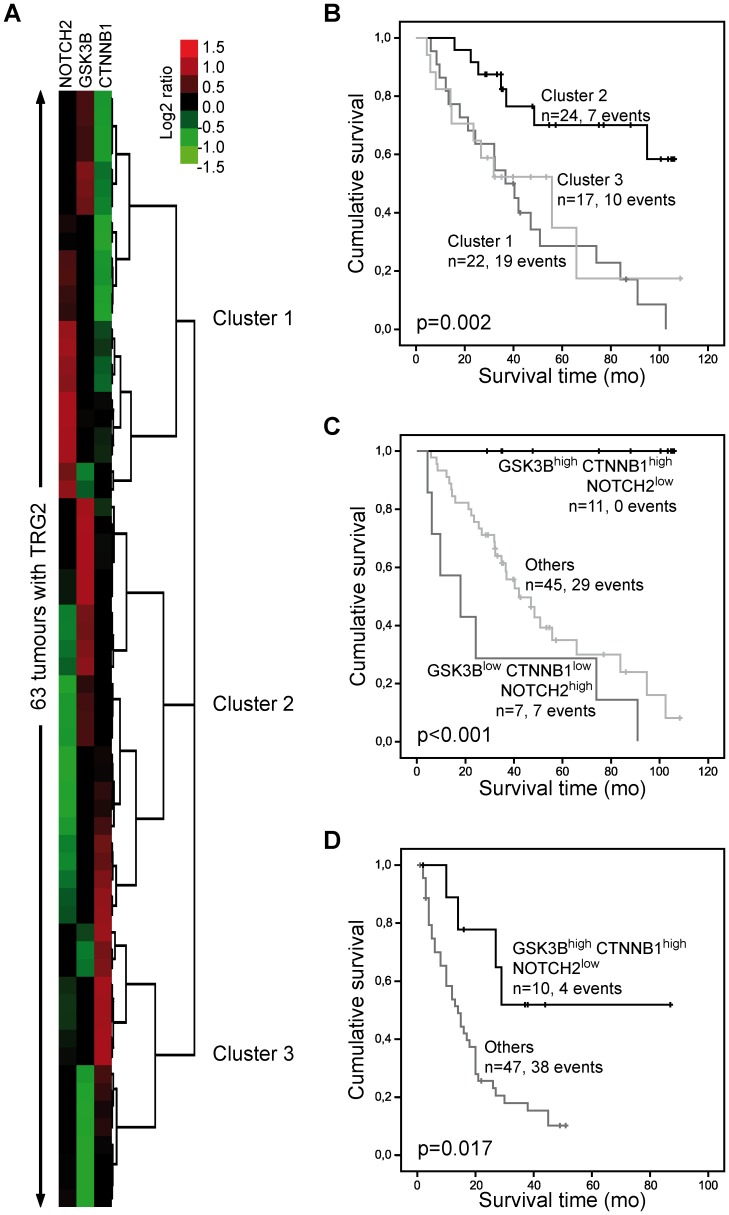
Expression of *GSK3B, CTNNB1* and *NOTCH2* and association with survival. A) Clustering of tumours based on expression of *GSK3B*, *CTNNB1* and *NOTCH2*. **B)** The Kaplan-Meier curves of the patient clusters show better survival of patients in cluster 2 (median OS not reached) compared to cluster 1 (median OS 36.7 mo, 95% CI 24.4–49.1) or cluster 3 (median OS 55.9 mo, 95% CI 16.7–95.0). **C)** Kaplan-Meier curves of patients based on the categorisation of tumours according to the optimal cut-off values for the three genes (*GSK3B* ^high^ *CTNNB1* ^high^ *NOTCH2* ^low^: median OS not reached; *GSK3B* ^low^ *CTNNB1* ^low^ *NOTCH2* ^high^: median OS 18.0 mo, 95% CI 0–39.5; Others: median OS 42.1 mo, 95% CI 28.3–55.9). **D)** Analysis of publically available array data of gastric cancer [32]. Kaplan-Meier curves of patients categorised according to the combined expression of *GSK3B*, *CTNNB1* and *NOTCH2* in the tumours using optimal cut-off values are shown (*GSK3B* ^high^ *CTNNB1* ^high^ *NOTCH2* ^low^: median OS not reached; Others: median OS 14.6 mo, 95% CI 8.6–19.3). P-values were determined by the log-rank test.

A cluster analysis encompassing all of the analysed genes revealed no patient groups that exhibited an association with OS. A cluster analysis of Wnt- and Notch signalling-associated genes produced the most significant association with OS when *GSK3B*, *CTNNB1* and *NOTCH2* were included (p = 0.002) (Figure 1A and B). According to the results of the cluster analysis, we grouped the patients into three groups with different combinations of high or low expression of *GSK3B*, *CTNNB1* and *NOTCH2*, which was defined by the optimal cut-off-values for gene expression in association with patient survival. The group with *GSK3B* ^high^, *CTNNB1* ^high^ and *NOTCH2* ^low^ expression showed the best survival, whereas the group with *GSK3B* ^low^, *CTNNB1* ^low^ and *NOTCH2* ^high^ expression had the worst overall outcome (p<0.001, Figure 1C).

Calculation of a risk score based on a multivariate Cox proportional hazard regression model of these three genes and dichotomisation of the patients according to the optimal cut-off value for OS into a high (n = 37) and low (n = 26) risk group demonstrated a statistically significant difference for OS (median survival of low and high risk patients: not reached and 37 months respectively; p<0.001). In addition, the difference in the respective survival rates at 1, 2, 3 and 5 years between the low and high risk group were statistically significant (Table S5).

### Differential Gene Expression Analysis between Corresponding Pre- and Post-therapeutic Tumour Samples

We next determined, whether the expression levels of the genes that exhibited a significant or moderate association with OS (p<0.1, Table 3 and 4) differ between pre-therapeutic biopsies and their corresponding post-therapeutic tumour specimen. Additionally, *POU5F1*, *LGR5* and *CCND1* were analysed, and tumour samples of patients with TRG2 or TRG3 (each n = 22) were studied.

**Table 4:** Gene expression and association with survival – univariate Cox regression analysis.

Gene	HR^1^	95% CI^2^	p-value
*GSK3B*	0.220	0.064–0.756	0.016
*DNMT1*	0.379	0.138–1.042	0.060
*CTNNB1*	0.567	0.305–1.052	0.072
*ABCG2*	0.851	0.707–1.024	0.087
*OLFM4*	0.987	0.969–1.006	0.191
*NOTCH2*	3.326	0.935–11.840	0.064

1 hazard ratio,

2 95% confidence interval.

In patients with TRG2, the expression of *NOTCH2*, *POU5F1* and *LGR5* increased significantly between the pre- and the post-therapeutic specimens (p = 0.002, 0.028 and 0.017, respectively) and the expression of *DNMT1* decreased (p = 0.009). In the group with TRG3, *POU5F1* exhibited a significant increase (p = 0.002), while *DNMT1* and *CCND1* significantly decreased (p = 0.002 and 0.007, respectively).

Regarding the expression of the prognostic-relevant genes *GSK3B* and *CTNNB1,* no statistically significant differences were observed between the pre- and the post-therapeutic tumour samples (Table 5 and Tables S2 and S3).

**Table 5:** Alterations of expression between pre- and post-therapeutic tumours of patients with tumour regression grade (TRG) 2 and 3.

	TRG2		TRG3	
Alteration	Gene	p-value^1^	Gene	p-value^1^
**Increase**				
	*NOTCH2*	0.002	*NOTCH2*	0.062
	*POU5F1*	0.028	*POU5F1*	0.002
	*LGR5*	0.017		
	*CTNNB1*	0.062		
**No change**				
	*ABCG2*	0.263	*LGR5*	0.249
	*GSK3B*	0.263	*CTNNB1*	0.733
	*OLFM4*	0.211	*ABCG2*	0.485
	*CCND1*	0.178	*GSK3B*	0.709
			*OLFM4*	0.961
**Decrease**				
	*DNMT1*	0.009	*CCND1*	0.007
			*DNMT1*	0.002

1 Wilcoxon signed rank test.

### Analysis of Publically Available Array Data

To evaluate the general prognostic value of the *GSK3B* ^high^, *CTNNB1* ^high^ and *NOTCH2* ^low^ expression pattern, we used a publically available expression array data set of gastric carcinomas [33]. The data set included the genes of interest and the relevant clinical information (OS, tumour stage) necessary to perform an analogous analysis. The determination of the optimal cut-off values of gene expression for correlation with survival and evaluation of the combined expression signature of *GSK3B* ^high^, *CTNNB1* ^high^ and *NOTCH2* ^low^ identified a group of 10 patients who had a significantly longer OS (p = 0.017, median OS: not reached) compared with the 47 remaining patients (median OS: 14.0 mo, 95% CI: 8.7–19.4) (Figure 1D).

Calculation of the risk score for this patient group and dichotomisation of the patients according to the optimal cut-off value for OS showed a considerably longer OS for the low risk group (n = 27) compared to the high risk group (n = 30), although the difference was not statistically significant (median survival 21 versus 13 months, p = 0.110). Considering the differences in the respective survival rates at 1, 2 and 3 years demonstrated analogous results with the most obvious difference at 2 years with a survival rate of 45% of the low risk patients and 21% of the high risk patients (p = 0.071) (Table S7).

### Immunohistochemical Analysis of NOTCH2

To evaluate the differences in gene expression between the paired pre- and post-therapeutic tumours on the protein level, we performed immunohistochemistry and focused on NOTCH2 (Figure S1B and C). We analysed the same patient groups that had been studied on the mRNA level. In addition, 21 patients with TRG1b and a control group of 16 patients treated by surgery alone were included.

**Figure 2:**
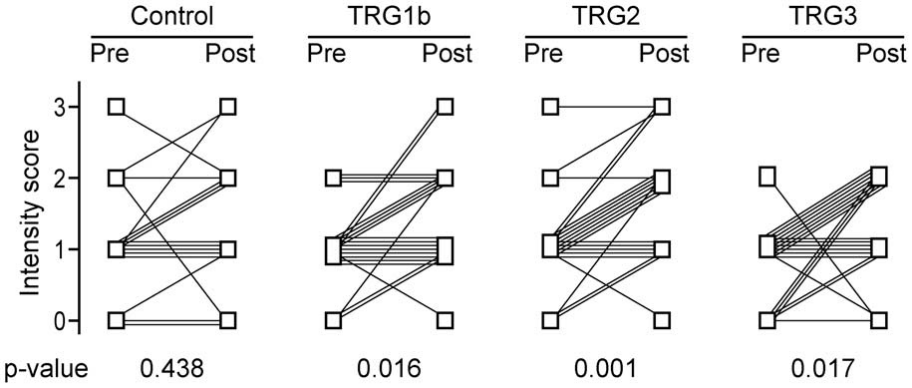
Alterations in the immunohistochemical staining for NOTCH2 between pre-therapeutic biopsies and their corresponding post-therapeutic tumours. Alterations of cytoplasmic staining intensities are shown. Each line indicates the alteration of the immunohistochemical staining score between the pre-therapeutic biopsy (Pre) and the corresponding post-therapeutic tumour specimen (Post) for each case. P-values were determined by the Wilcoxon signed rank test (exact).

A comparison of cytoplasmic staining intensities between pre-therapeutic biopsies and their corresponding post-therapeutic tumours revealed a statistically significant increase in staining intensity in the post-therapeutic specimens from patients with TRG1b, 2 and 3 (p = 0.016, 0.001, and 0.017, respectively). In contrast, no differences were observed in patients treated by surgery alone (p = 0.438) (Figure 2). The percentage of stained cells was not significantly altered. Regarding nuclear staining, a significant decrease in staining intensity in the post-therapeutic tumour specimen was found in the group with TRG2 (p = 0.007), TRG3 (p = 0.015) and in the control group not treated by chemotherapy (p = 0.016). A significant decrease in the percentage of cells with stained nuclei was observed in the group with TRG1b (p = 0.005), TRG2 (p<0.001), TRG3 (p = 0.003) as well as in the control group (p = 0.001).

## Discussion

Our study analysing the expression of CSC related genes in residual gastric cancer cells after neoadjuvant chemotherapy identified a gene signature with a high prognostic impact composed of *GSK3B*, the *β-catenin* gene *CTNNB1 and NOTCH2*. Interestingly, high expression levels of *CTNNB1* and *GSK3B* were associated with increased survival. Β-catenin is a key molecule in the transmission of Wnt signalling to the nucleus and drives multiple cellular processes [35]. Aberrant Wnt signalling has been demonstrated in up to 46% of GCs, and both Wnt/*β-catenin* and proliferation/stem cell expression signatures indicating the respective pathway activation, were associated with decreased patient survival [36]. *CTNNB1* mRNA levels can modulate Wnt signalling [37], and Wnt activity has been linked to cancer stemness in the colon [38]. Given these data, a negative association of *CTNNB1* expression in residual tumour cells after chemotherapy and the survival of the patients would have been expected. However, *β*-catenin is also part of the E-cadherin-catenin complex and alterations in this cell adhesion complex have been associated with worse prognosis in various tumours [39]. In GC, conflicting results regarding the association between *β*-catenin expression and patient prognosis exist [40], [41].

It is important to note, that GSK3B is also a multifunctional protein involved in various signalling networks and antagonises Wnt signalling by mediating the degradation of *β*-catenin, although a Wnt-activating function has also been described [42]–[44]. Increased expression of *GSK3B* was associated with a favourable prognosis in our study, which is consistent with a report of higher *GSK3B* mRNA expression associated with better survival in ovarian high-grade serous carcinomas [45].

Higher *NOTCH2* expression levels correlated with worse survival in our GC patients. The Notch receptor family encompasses four members, and NOTCH1 and NOTCH2 have been implicated to enhance gastric cancer progression [46], [47]. Furthermore, an association of NOTCH1 expression with poor prognosis has been reported [48], which is not consistent with our findings; however, due to the differences in the study populations, these results are not directly comparable.

Given the highly significant association of the gene signature encompassing *CTNNB1*, *GSK3B* and *NOTCH2* with patient survival and the significant risk score–based classification of the patients into a high and low risk group, we were particularly interested, if this were related to chemotherapy. The comparison of *GSK3B* and *CTNNB1* expression levels between pre- and post-therapeutic tumour samples revealed no clear differences, whereas a significant increase in the expression of *NOTCH2* was found. This finding suggests that *CTNNB1* and *GSK3B* expression may reflect a property of the primary tumour that is not altered by chemotherapy and that *NOTCH2* expression in the residual tumour cells is at least partly related to this treatment.

An analysis of the prognostic significance of the three-genes in publically available genome-wide expression data of advanced GC demonstrated a significant association for the specific gene expression pattern of *GSK3B* ^high^, *CTNNB1* ^high^ and *NOTCH2* ^low^ expression and increased patient survival and a similar tendency considering the risk score-based classification.

This finding supports the interpretation that the prognostic effect observed in our study mainly reflects a property of the primary gastric tumour, suggesting a critical role for these genes in the biology of these tumours. In addition, the prognostic effect observed might be enhanced by an increase in the expression of *NOTCH2* in the residual tumour after chemotherapy. Thus, if validated in a prospective study, this three gene signature might be useful for risk stratification of GC patients and additionally may guide postoperative treatment after neoadjuvant chemotherapy.

Our results are reminiscent of a recent study analysing a CSC-derived gene signature that predicts tumour recurrence in the colon and demonstrates that the elevated expression of Wnt target genes is indicative of a favourable prognosis [48]. The authors provide evidence that this association more likely reflects the differentiation status of the malignant tissue rather than the number of CSCs [49].

In considering the alterations of *NOTCH2* expression between corresponding pre- and post-therapeutic tumours, it is important to note that comparing gene expression at the mRNA-level, cannot distinguish whether these alterations reflect a relative enrichment of the cells expressing this gene, whether they are due to the chemotherapeutic agents affecting gene transcription in the cells *per se*, or whether the alterations reflect mere sampling differences. To clarify this issue we analysed NOTCH2 protein expression by immunohistochemistry and included tumours from patients treated by surgery alone. Based on the cytoplasmic staining, our results confirm an increase in NOTCH2 expression at the protein level in the post-therapeutic tumours and they demonstrate that the observed differences are likely to be restricted to patients treated by chemotherapy. Of note, the increase in NOTCH2 expression was related to an increase in the cytoplasmic staining intensity rather than to an increase in the number of cells expressing NOTCH2. This result argues against an enrichment of a subpopulation of NOTCH2-expressing tumour cells and more likely suggests a chemotherapy-induced increase in gene expression in the tumour cells, which may be related to the tumour biological features after neoadjuvant treatment. However, a clear distinction between these possibilities may be limited by the semiquantitative evaluation of immunohistochemical staining. As similar alterations in nuclear staining were observed in all tumour groups including the control we considered these changes as unrelated to chemotherapy.

Irrespective of the mechanism and the true nature of the residual tumour cells expressing *NOTCH2*, our results may have therapeutic implications. Notch signalling has emerged as a potential new therapeutic target, and gamma-secretase inhibitors, which inhibit the processing of the Notch receptors, are currently being evaluated in clinical trials [50]. Our study suggests that targeting Notch signalling may also represent a new strategy to treat GC patients. As an adverse prognostic effect was only associated with *NOTCH2* and not *NOTCH1*, our data also indicate that a detailed characterisation of the individual Notch receptors and a thorough functional investigation are mandatory and further strongly favour the development of Notch paralog-specific inhibitory agents.

A significant increase in *POU5F1* expression was observed after chemotherapy in the resected specimens in our study. The POU5F1 transcription factor is essential for the maintenance of self-renewal, and its high expression in residual cancer cells after radiochemotherapy is correlated with poor prognosis in colon cancer [51]. Interestingly we also observed an increased expression of *LGR5*, a promising intestinal CSC marker, after chemotherapy in tumours with TRG2 [18]. These results are compatible with the potential enrichment of drug-resistant tumour cells expressing *POU5F1* or *LGR5*, but the underlying mechanism for these alterations and the particular properties of the cells expressing these genes remain to be determined.

In our study, no association with survival were observed for the cell surface molecules CD44 or CD133, both of which have been widely used to identify putative CSCs in various tumours [4]–[11]. This result supports recent findings demonstrating that these cell surface molecules do not identify CSCs in primary gastric tumours [20].

Taken together, our findings demonstrate that the expression signature of *GSK3B* ^high^, *CTNNB1* ^high^ and *NOTCH2* ^low^ in chemotherapy-resistant residual GC tumour cells is a strong predictor for favourable patient prognosis. This prognostic relevance was also demonstrated for GC patients using publically available gene expression data. The results of the differential expression analysis of the pre- and post-therapeutic tumour specimen also suggests that the impact of *GSK3B* and *CTNNB1* to this signature is not dependent on chemotherapy but rather related to a property of the primary tumour. They further indicate a prominent role for NOTCH2 and chemotherapy resistance in GC, which is more likely related to an effect of the chemotherapeutic agents on *NOTCH2* expression rather than to an enrichment of *NOTCH2* expressing tumour cells.

## Supporting Information

Figure S1 Western blot and immunohistochemistry with the anti-NOTCH2 antibody. **A)** The antibody directed against the NOTCH2 intracellular domain specifically detects the full length NOTCH2 protein above the 250 kDa marker as well as the cleaved forms NOTCH Extracellular Truncated (NEXT) and NOTCH Intracellular Domain (NICD) at approximately 110 kDa. **B)** A weak cytoplasmic immunohistochemical staining in the pre-therapeutic biopsy sample and **C)** a strong cytoplasmic staining in the corresponding post-therapeutic tumour with TRG2 is shown. Scale bars indicate 50 µm.
(TIF)

Table S1 Multivariate Cox regression analysis. Gene expression of *GSK3B, CTNNB1, DNMT1* and the standard prognostic variables in GC, ypT, ypN, ypM and resection category were included in the model.
(DOC)

Table S2 Gene expression data of the pre- and corresponding post-therapeutic tumour samples of patients with TRG2.
(DOC)

Table S3 Gene expression data of the pre- and corresponding post-therapeutic tumour samples of patients with TRG3.
(DOC)

Table S4 Multivariate Cox regression data for the own dataset.
(DOC)

Table S5 Relative survival rates based on the dichotomised risk score (own data).
(DOC)

Table S6 Multivariate Cox regression data for the publically available dataset.
(DOC)

Table S7 Relative survival rates based on the dichotomised risk score (public data).
(DOC)

Material and Methods S1 Information on experimental details on quantitative real time PCR, immunohistochemistry, Western blotting, the analysis of public microarray data and multivariate Cox regression based risk scores.
(DOC)
