# Supplementary figures and images for: Expression Profiling of Stem Cell-Related Genes in Neoadjuvant-Treated Gastric Cancer: A NOTCH2, GSK3B and β-catenin Gene Signature Predicts Survival
Source: PLoS One. 2012 Sep 10;7(9):e44566. doi: 10.1371/journal.pone.0044566 (PMC3438181; doi:10.1371/journal.pone.0044566)

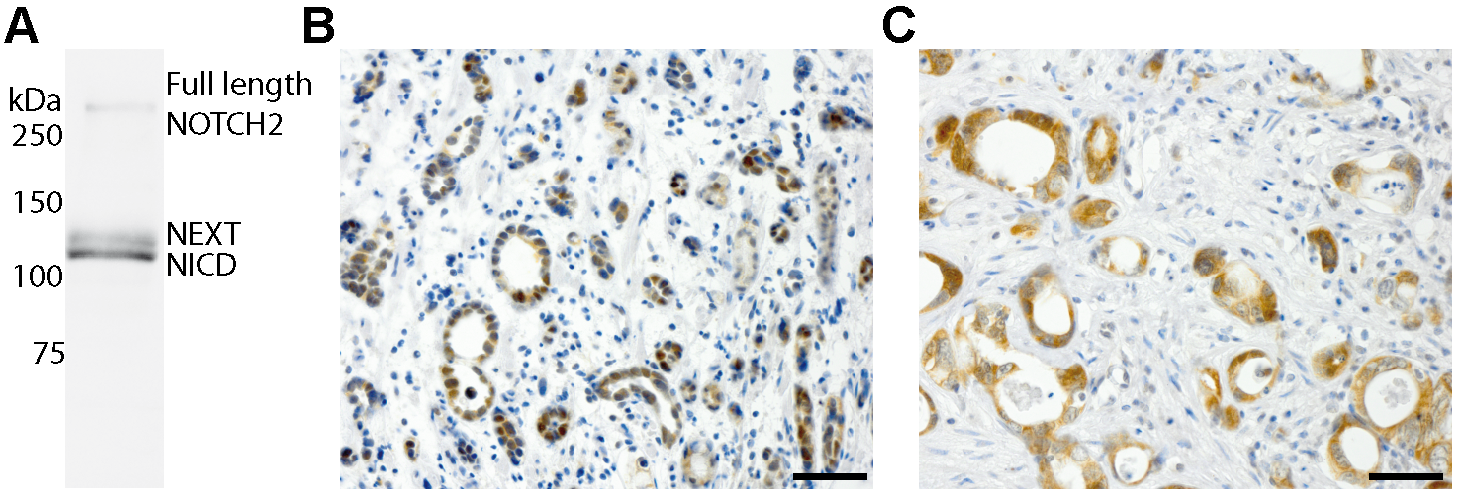

Supplement: Figure S1 — Western blot and immunohistochemistry with the anti-NOTCH2 antibody. A) The antibody directed against the NOTCH2 intracellular domain specifically detects the full length NOTCH2 protein above the 250 kDa marker as well as the cleaved forms NOTCH Extracellular Truncated (NEXT) and NOTCH Intracellular Domain (NICD) at approximately 110 kDa. B) A weak cytoplasmic immunohistochemical staining in the pre-therapeutic biopsy sample and C) a strong cytoplasmic staining in the corresponding post-therapeutic tumour with TRG2 is shown. Scale bars indicate 50 µm. (TIF) [file pone.0044566.s001.tif]
