# Supplementary material for: Expression Profiling of Stem Cell-Related Genes in Neoadjuvant-Treated Gastric Cancer: A NOTCH2, GSK3B and β-catenin Gene Signature Predicts Survival
Source: PLoS One. 2012 Sep 10;7(9):e44566. doi: 10.1371/journal.pone.0044566 (PMC3438181; doi:10.1371/journal.pone.0044566)
Supplement: Table S1 — Multivariate Cox regression analysis. Gene expression of GSK3B, CTNNB1, DNMT1 and the standard prognostic variables in GC, ypT, ypN, ypM and resection category were included in the model. (DOC) [file pone.0044566.s002.doc]

**Table S1: Multivariate Cox Regression Analysis^1^**

| Rank | Variable | HR^2^ | 95% CI^3^ | p-value |
| --- | --- | --- | --- | --- |
| 1 | distant metastasis | 23.893 | 8.159 – 69.972 | <0.001 |
| 2 | *GSK3B* | 0.128 | 0.033 – 0.492 | 0.003 |

^1^forward selection (likelihood ratio), candidate parameters are *GSK3B*, *DNMT1* and *CTNNB1* expression, ypT, ypN, ypM and resection-category. ^2^hazard ratio, ^3^95% confidence interval
