# Supplementary material for: Expression Profiling of Stem Cell-Related Genes in Neoadjuvant-Treated Gastric Cancer: A NOTCH2, GSK3B and β-catenin Gene Signature Predicts Survival
Source: PLoS One. 2012 Sep 10;7(9):e44566. doi: 10.1371/journal.pone.0044566 (PMC3438181; doi:10.1371/journal.pone.0044566)
Supplement: Table S2 — Gene expression data of the pre- and corresponding post-therapeutic tumour samples of patients with TRG2. (DOC) [file pone.0044566.s003.doc]

**Table S2: Gene Expression Data^1^ in Pre- and Post-therapeutic Tumours of Patients with TRG2**

| Tumour | *ABCG2* | | *CCND1* | | *CTNNB1* | | *DNMT1* | | *GSK3B* | | *LGR5* | | *NOTCH2* | | *OLFM4* | | *POU5F1* | |
| --- | --- | --- | --- | --- | --- | --- | --- | --- | --- | --- | --- | --- | --- | --- | --- | --- | --- | --- |
|  | Pre | Post | Pre | Post | Pre | Post | Pre | Post | Pre | Post | Pre | Post | Pre | Post | Pre | Post | Pre | Post |
| 201 | 0.8074 | 0.6552 | 0.3112 | 1.2135 | 1.4209 | 3.3176 | 0.455 | 0.0874 | 0.9723 | 1.6392 | 101.7243 | 148.5731 | 0.4766 | 1.5522 | 0.762 | 0.1458 | 1.2494 | 1.4191 |
| 202 | 0.0852 | 0.4613 | 0.8503 | 0.2967 | 0.6282 | 0.5451 | 0.7019 | 0.9919 | 1.2142 | 0.7929 | 0.6034 | 17.6483 | 1.4694 | 1.7401 | 0.0542 | 267.6109 | 0.1434 | 1.4015 |
| 203 | 1.2383 | 1.8214 | 0.3146 | 0.1961 | 0.7502 | 0.8685 | 0.323 | 0.5671 | 0.8219 | 0.6525 | 51.659 | 3.9456 | 0.8601 | 0.8016 | 129.657 | 27.6771 | 0.2765 | 2.3446 |
| 204 | 0.1014 | 0.3328 | 0.2411 | 0.2585 | 0.2857 | 0.3493 | 0.7288 | 0.6158 | 0.7721 | 0.6505 | 0.6282 | 0.7479 | 0.8485 | 1.4427 | 692.4945 | 315.0644 | 0.1706 | 0.7565 |
| 205 | 0.0405 | 0.7312 | 0.219 | 0.2769 | 0.886 | 0.8171 | 0.6359 | 0.3341 | 1.2286 | 1.1827 | 1.4197 | 2.4436 | 0.8447 | 1.4966 | 383.8265 | 81.4221 | 2.0206 | 0.9861 |
| 206 | 0.0497 | 0.3178 | 1.1713 | 0.7172 | 1.5681 | 2.055 | 0.5028 | 0.1962 | 1.4827 | 0.8467 | 75.4639 | 177.2097 | 0.4197 | 0.7998 | 0.0687 | 0.6154 | 0.769 | 1.4964 |
| 207 | 0.8987 | 0.4693 | 0.7428 | 1.9114 | 0.6652 | 1.4285 | 0.422 | 0.1895 | 1.2888 | 1.2944 | 1.7902 | 7.0128 | 0.5183 | 0.8632 | 1119.2955 | 0.0287 | 1.084 | 1.5989 |
| 208 | 0.0706 | 1.4756 | 0.1279 | 0.3109 | 0.5038 | 1.28 | 0.6106 | 0.2842 | 0.5846 | 0.9157 | 71.6252 | 1.5105 | 0.6412 | 0.3202 | 1.1398 | 232.3512 | 3.6831 | 0.6463 |
| 209 | 0.1996 | 1.7917 | 0.3831 | 0.2834 | 1.1201 | 0.9718 | 0.5558 | 0.4597 | 1.4717 | 1.0303 | 6.9964 | 16.8946 | 0.7392 | 0.8522 | 231.7455 | 258.4982 | 1.2324 | 2.0104 |
| 210 | 2.5151 | 2.0767 | 0.2986 | 0.1284 | 1.0661 | 1.0229 | 0.5988 | 0.5219 | 1.3518 | 1.2476 | 2.3114 | 4.8295 | 1.1176 | 1.455 | 223.5681 | 10.3371 | 1.6759 | 3.7562 |
| 211 | 0.2699 | 0.3584 | 0.438 | 0.251 | 1.101 | 0.9026 | 0.4407 | 0.5325 | 1.1956 | 1.3331 | 5.4436 | 34.1209 | 0.8212 | 1.5435 | 0.0347 | 0.0501 | 0.628 | 1.0365 |
| 212 | 0.6549 | 1.5608 | 0.3766 | 0.2526 | 1.1749 | 1.4098 | 0.6057 | 0.4664 | 1.2964 | 2.1193 | 22.2134 | 5.8071 | 1.0469 | 3.9938 | 0.987 | 0.2292 | 0.2794 | 13.5824 |
| 213 | 0.9174 | 0.0841 | 0.1519 | 0.2183 | 1.2051 | 1.0704 | 0.6669 | 0.4584 | 1.4991 | 1.484 | 26.7312 | 113.4485 | 0.7029 | 1.5945 | 10.3161 | 70.2802 | 0.6732 | 3.2178 |
| 214 | 0.0552 | 2.3656 | 0.3118 | 0.2617 | 0.9801 | 1.6162 | 0.5223 | 0.2907 | 2.1854 | 2.3502 | 1.935 | 54.4139 | 0.7852 | 0.3777 | 163.5909 | 0.1199 | 1.0316 | 2.3454 |
| 215 | 0.0371 | 1.0321 | 0.4079 | 0.361 | 1.1549 | 2.2331 | 0.4106 | 0.2463 | 1.09 | 1.3605 | 30.2997 | 683.4151 | 0.9404 | 0.8177 | 5.4579 | 0.6407 | 0.7017 | 0.8593 |
| 216 | 1.9428 | 0.2155 | 0.616 | 1.0875 | 0.7839 | 1.682 | 0.2456 | 0.3512 | 1.4702 | 1.3149 | 10.8778 | 39.5049 | 0.9319 | 1.0968 | 11.6086 | 0.0313 | 13.1581 | 1.5536 |
| 217 | 1.6709 | 0.8923 | 0.2135 | 0.1513 | 0.5315 | 0.9231 | 0.7599 | 0.5363 | 1.3805 | 0.9901 | 1.0148 | 54.4469 | 1.0391 | 1.5276 | 12.5184 | 75.921 | 0.5238 | 0.8343 |
| 218 | 0.6033 | 3.5607 | 0.595 | 0.4215 | 1.6405 | 1.5742 | 0.7806 | 0.7051 | 1.0301 | 0.9656 | 0.8667 | 43.2526 | 0.9701 | 1.5433 | 58.0892 | 11.4705 | 0.4093 | 2.4773 |
| 219 | 1.1786 | 1.0894 | 1.2613 | 0.7009 | 0.9266 | 0.976 | 0.6216 | 0.6257 | 1.2923 | 1.1169 | 1.045 | 144.7606 | 0.7611 | 0.8415 | 1.3093 | 1.0458 | 0.4256 | 1.685 |
| 220 | 1.4797 | 1.622 | 0.2774 | 0.24 | 0.8511 | 0.952 | 0.5503 | 0.3354 | 0.9571 | 0.8777 | 33.7736 | 1.9235 | 0.7069 | 1.6548 | 36.9037 | 1.4824 | 0.6087 | 1.0209 |
| 221 | 1.0000 | 0.6255 | 1.0000 | 0.5268 | 1.0000 | 0.939 | 1.0000 | 0.6079 | 1.0000 | 0.8571 | 1.0000 | 156.9554 | 1.0000 | 1.1155 | 1.0000 | 0.6648 | 1.0000 | 0.5732 |
| 222 | 1.5372 | 1.0089 | 1.0019 | 0.1499 | 1.2521 | 0.9053 | 0.4616 | 0.4052 | 1.5289 | 1.0529 | 5.1637 | 1.2515 | 1.3582 | 1.3651 | 16.5221 | 48.2165 | 0.5207 | 1.3103 |

^1^Relative quantification (RQ)-data normalised to geometric mean of POLR2A, IPO8 and UBC, Pre: values for pre-therapeutic biopsies, Post: values for post-therapeutic tumours
