# Supplementary material for: Expression Profiling of Stem Cell-Related Genes in Neoadjuvant-Treated Gastric Cancer: A NOTCH2, GSK3B and β-catenin Gene Signature Predicts Survival
Source: PLoS One. 2012 Sep 10;7(9):e44566. doi: 10.1371/journal.pone.0044566 (PMC3438181; doi:10.1371/journal.pone.0044566)
Supplement: Table S3 — Gene expression data of the pre- and corresponding post-therapeutic tumour samples of patients with TRG3. (DOC) [file pone.0044566.s004.doc]

**Table S3: Gene Expression Data^1^ in Pre- and Post-therapeutic Tumours of Patients with TRG3**

| Tumour | *ABCG2* | | *CCND1* | | *CTNNB1* | | *DNMT1* | | *GSK3B* | | *LGR5* | | *NOTCH2* | | *OLFM4* | | *POU5F1* | |
| --- | --- | --- | --- | --- | --- | --- | --- | --- | --- | --- | --- | --- | --- | --- | --- | --- | --- | --- |
|  | Pre | Post | Pre | Post | Pre | Post | Pre | Post | Pre | Post | Pre | Post | Pre | Post | Pre | Post | Pre | Post |
| 301 | 1.2813 | 0.4502 | 0.775 | 0.5687 | 0.9389 | 0.6044 | 0.5527 | 0.1193 | 0.9329 | 0.5342 | 2.4776 | 1.3538 | 0.7759 | 0.795 | 11.2133 | 520.5333 | 0.5137 | 1.5234 |
| 302 | 0.3885 | 0.4456 | 1.1229 | 0.8355 | 0.686 | 0.8155 | 0.6625 | 0.2967 | 1.693 | 1.3639 | 36.6264 | 54.6226 | 0.6943 | 0.9173 | 26.3585 | 501.7576 | 1.1401 | 1.9515 |
| 303 | 0.2844 | 0.3496 | 0.4626 | 0.3638 | 1.42 | 1.1997 | 1.1346 | 1.2031 | 1.3085 | 0.9831 | 49.2732 | 13.5616 | 0.5684 | 0.2633 | 200.394 | 51.0116 | 0.6845 | 1.5658 |
| 304 | 0.0561 | 0.0426 | 0.9735 | 0.4503 | 1.0421 | 0.5369 | 0.4042 | 1.0378 | 0.7275 | 0.9633 | 1.9529 | 1.4843 | 0.7388 | 0.3279 | 0.6005 | 0.0586 | 19.3556 | 1.3932 |
| 305 | 0.0386 | 0.036 | 0.202 | 0.4287 | 0.5224 | 0.6038 | 0.8181 | 0.3779 | 0.7839 | 0.5309 | 1.3457 | 1.2546 | 0.5203 | 0.6491 | 164.0997 | 28.0927 | 0.9104 | 1.2465 |
| 306 | 0.4164 | 1.2608 | 0.3364 | 0.3072 | 0.8242 | 0.805 | 0.8796 | 0.6998 | 0.859 | 0.9324 | 1.1344 | 58.8919 | 0.4324 | 0.5237 | 16.3279 | 0.7477 | 0.794 | 0.6184 |
| 307 | 0.0889 | 0.8219 | 0.2791 | 0.1605 | 0.4939 | 0.4783 | 0.3397 | 0.3731 | 0.7225 | 1.1404 | 3.0949 | 44.8285 | 0.3754 | 0.927 | 97.7846 | 1.1848 | 1.5844 | 1.219 |
| 308 | 0.0976 | 0.7237 | 0.3165 | 0.234 | 0.445 | 0.5208 | 0.5634 | 0.4726 | 0.8192 | 0.9633 | 1.5598 | 26.6483 | 0.5606 | 1.2625 | 501.9979 | 1436.0642 | 0.8566 | 1.4577 |
| 309 | 0.0433 | 0.5004 | 1.6499 | 0.3241 | 0.5891 | 0.5455 | 0.3982 | 0.3399 | 1.1794 | 1.1095 | 1.5064 | 0.9972 | 1.4218 | 1.2131 | 9.7911 | 58.1104 | 1.0889 | 1.1174 |
| 310 | 0.4311 | 0.0478 | 0.3008 | 0.1923 | 0.5286 | 0.6896 | 0.6167 | 0.4183 | 1.273 | 1.2639 | 19.4233 | 61.7708 | 0.5875 | 1.0459 | 1488.5276 | 69.5029 | 0.2259 | 1.4073 |
| 311 | 0.4427 | 0.2258 | 0.1914 | 0.2742 | 0.571 | 0.6374 | 0.259 | 0.1799 | 0.9458 | 0.6739 | 12.0448 | 5.8125 | 0.4726 | 0.6112 | 353.3179 | 6.6043 | 0.4101 | 1.4246 |
| 312 | 0.5838 | 0.7062 | 0.2521 | 0.1447 | 0.6681 | 0.9669 | 0.4101 | 0.3444 | 1.0564 | 1.25 | 21.7974 | 1.6856 | 0.3992 | 1.1266 | 57.2789 | 1092.3336 | 0.433 | 1.0214 |
| 313 | 0.6306 | 0.557 | 0.4903 | 0.3736 | 0.7881 | 1.0328 | 0.5953 | 0.2684 | 0.8636 | 0.7036 | 54.6242 | 63.3359 | 1.0111 | 1.0951 | 13.9233 | 2.2728 | 0.7303 | 1.2849 |
| 314 | 0.1973 | 0.2384 | 0.4612 | 0.446 | 0.7843 | 1.4012 | 0.7996 | 0.548 | 1.3626 | 1.3166 | 0.7729 | 59.8122 | 0.7634 | 1.1866 | 27.7366 | 19.1438 | 0.6482 | 1.4504 |
| 315 | 0.0379 | 0.0409 | 0.5117 | 0.4221 | 0.9071 | 0.8848 | 0.6998 | 0.2507 | 0.8277 | 1.0193 | 14.1271 | 1.4235 | 0.7976 | 0.9068 | 44.9336 | 0.0562 | 0.6753 | 0.9977 |
| 316 | 0.4266 | 0.4754 | 0.3953 | 0.5037 | 1.2607 | 1.1648 | 0.7194 | 0.7252 | 0.9394 | 0.7742 | 10.9794 | 14.8472 | 0.4421 | 0.5366 | 1960.7413 | 298.5127 | 0.7005 | 1.4496 |
| 317 | 3.7058 | 10.9631 | 0.4031 | 0.2677 | 0.9599 | 0.9859 | 0.5902 | 0.5503 | 1.0415 | 1.3849 | 89.6942 | 147.5321 | 0.7581 | 0.5502 | 354.114 | 792.6166 | 0.1395 | 0.7721 |
| 318 | 0.2702 | 0.0573 | 0.3251 | 0.2159 | 1.4606 | 1.1051 | 0.6117 | 0.5323 | 0.6028 | 0.966 | 46.3734 | 1.9953 | 0.683 | 0.7811 | 71.8938 | 77.8421 | 0.4768 | 2.1143 |
| 319 | 0.958 | 0.4146 | 0.9157 | 0.6309 | 1.3612 | 1.0575 | 1.1339 | 0.342 | 1.0667 | 0.918 | 100.8472 | 56.7456 | 0.8197 | 0.9973 | 0.2003 | 0.0693 | 1.9446 | 4.0389 |
| 320 | 0.2311 | 0.3624 | 0.0939 | 0.0924 | 0.4389 | 0.5295 | 1.8174 | 0.547 | 1.1252 | 1.12 | 0.7071 | 7.158 | 0.3535 | 0.5204 | 6.0994 | 48.4571 | 0.3013 | 0.8798 |
| 321 | 0.3355 | 0.606 | 0.3035 | 0.4092 | 0.5046 | 1.0311 | 0.5927 | 0.3469 | 0.8103 | 0.9929 | 1.6217 | 17.4013 | 0.6472 | 0.9995 | 2063.6932 | 4896.3743 | 0.5035 | 2.3099 |
| 322 | 1.2608 | 0.0405 | 0.1181 | 0.0864 | 0.9316 | 0.8983 | 0.7354 | 0.5462 | 1.4893 | 0.9542 | 1.0601 | 10.0897 | 0.8481 | 0.6205 | 15.586 | 4.1619 | 0.6587 | 1.4837 |

^1^Relative quantification (RQ)-data normalised to geometric mean of POLR2A, IPO8 and UBC, Pre: values for pre-therapeutic biopsies, Post: values for post-therapeutic tumours
