# Supplementary material for: Expression Profiling of Stem Cell-Related Genes in Neoadjuvant-Treated Gastric Cancer: A NOTCH2, GSK3B and β-catenin Gene Signature Predicts Survival
Source: PLoS One. 2012 Sep 10;7(9):e44566. doi: 10.1371/journal.pone.0044566 (PMC3438181; doi:10.1371/journal.pone.0044566)
Supplement: Table S4 — Multivariate Cox regression data for the own dataset. (DOC) [file pone.0044566.s005.doc]

**Table S4: Multivariate Cox regression data for the own dataset**

| **Gene** | **Coefficient** | **HR^1^** | **SE^2^** |
| --- | --- | --- | --- |
| CTNNB1 | -0.1913 | 0.8259 | 0.3273 |
| GSK3B | -1.5684 | 0.2084 | 0.7008 |
| NOTCH2 | 1.2036 | 3.3321 | 0.6371 |

^1^hazard ratio, ^2^standard error
