# Supplementary material for: Expression Profiling of Stem Cell-Related Genes in Neoadjuvant-Treated Gastric Cancer: A NOTCH2, GSK3B and β-catenin Gene Signature Predicts Survival
Source: PLoS One. 2012 Sep 10;7(9):e44566. doi: 10.1371/journal.pone.0044566 (PMC3438181; doi:10.1371/journal.pone.0044566)
Supplement: Table S5 — Relative survival rates based on the dichotomised risk score (own data). (DOC) [file pone.0044566.s006.doc]

**Table S5: Relative survival rates based on the** dichotomised risk score (own data)

|  | **1 year survival** | **2 year survival** | **3 year survival** | **5 year survival** |
| --- | --- | --- | --- | --- |
| **Score** | **number/total number of patients (%)** | **number/total number of patients (%)** | **number/total number of patients (%)** | **number/total number of patients (%)** |
| ≤ ‑1.3199^1^ | 26/26 (100 %) | 25/26 (96 %) | 16/20 (80 %) | 11/17 (65 %) |
| > ‑1.3199 | 31/37 (84 %) | 23/37 (62 %) | 16/34 (47 %) | 5/29 (17 %) |
| **p-value^2^** | 0.038 | 0.002 | 0.017 | 0.001 |

^1^optimal cut-point determined by log-rank statistics, ^2^Pearson’s chi-square test
