# Supplementary material for: Expression Profiling of Stem Cell-Related Genes in Neoadjuvant-Treated Gastric Cancer: A NOTCH2, GSK3B and β-catenin Gene Signature Predicts Survival
Source: PLoS One. 2012 Sep 10;7(9):e44566. doi: 10.1371/journal.pone.0044566 (PMC3438181; doi:10.1371/journal.pone.0044566)
Supplement: Table S6 — Multivariate Cox regression data for the publically available dataset. (DOC) [file pone.0044566.s007.doc]

**Table S6: Multivariate Cox regression data for the** publically available dataset

| **Gene** | **Coefficient** | **HR^1^** | **SE^2^** |
| --- | --- | --- | --- |
| CTNNB1 | -0.1496 | 0.8610 | 0.2117 |
| GSK3B | -0.2009 | 0.8180 | 0.2859 |
| NOTCH2 | 0.1005 | 1.1058 | 0.1500 |

^1^hazard ratio, ^2^standard error
