# Supplementary material for: Expression Profiling of Stem Cell-Related Genes in Neoadjuvant-Treated Gastric Cancer: A NOTCH2, GSK3B and β-catenin Gene Signature Predicts Survival
Source: PLoS One. 2012 Sep 10;7(9):e44566. doi: 10.1371/journal.pone.0044566 (PMC3438181; doi:10.1371/journal.pone.0044566)
Supplement: Table S7 — Relative survival rates based on the dichotomised risk score (public data). (DOC) [file pone.0044566.s008.doc]

**Table S7: Relative survival rates based on the dichotomised risk score (public data)**

|  | **1 year survival** | **2 year survival** | **3 year survival** |
| --- | --- | --- | --- |
| **Score** | **number/total number of patients (%)** | **number/total number of patients (%)** | **number/total number of patients (%)** |
| ≤ ‑1.3199^1^ | 16/23 (70 %) | 10/22 (45 %) | 7/22 (32 %) |
| > ‑1.3199 | 15/29 (52 %) | 6/28 (21 %) | 4/28 (14 %) |
| **p-value^2^** | 0.193 | 0.071 | 0.178 |

^1^optimal cut-point determined by log-rank statistics, ^2^Pearson’s chi-square and Fisher’s exact test
