# Supplementary material for: Expression Profiling of Stem Cell-Related Genes in Neoadjuvant-Treated Gastric Cancer: A NOTCH2, GSK3B and β-catenin Gene Signature Predicts Survival
Source: PLoS One. 2012 Sep 10;7(9):e44566. doi: 10.1371/journal.pone.0044566 (PMC3438181; doi:10.1371/journal.pone.0044566)
Supplement: Material and Methods S1 — Information on experimental details on quantitative real time PCR, immunohistochemistry, Western blotting, the analysis of public microarray data and multivariate Cox regression based risk scores. (DOC) [file pone.0044566.s009.doc]

#### Supporting Information

Supplementary Material and Methods

**Quantitative real time PCR**

Quantitative real time PCR was performed using the TaqMan® Universal PCR Master Mix and the ABI PRISM 7700 Sequence Detection System instrument (Applied Biosystems Inc., Foster City, CA). The cycling conditions included two initial steps at 50.0 °C for 2 min and 94.5 °C for 10 min and subsequently 40 cycles of 97.0 °C for 30 s and 59.7 °C for 1 min. The screening analysis of the 44 genes in the post-therapeutic tumours was performed in duplicate. The differential expression analysis between the corresponding pre-therapeutic biopsies and post-therapeutic specimen was performed in triplicates. SDS2.3, RQ Manager 1.2 and Data Assist v2.0 software (Applied Biosystems Inc., Foster City, CA) were used for data analysis.

##### Immunohistochemistry

The monoclonal antibody against NOTCH2 (C651.6DbHN), developed by Mr. Artavanis-Tsakonas [1], was obtained from the Developmental Studies Hybridoma Bank, developed under the auspices of the NICHD and maintained by The University of Iowa, Department of Biology, Iowa City, IA 52242. 2 µm sections of FFPE tissue were deparaffinised and subjected to heat-induced antigen retrieval in 10 mM citric acid buffer, pH 6. The endogenous peroxidase activity was blocked by incubation with 3 % H_2_O_2_ for 10 min. Nonspecific antibody binding was blocked in 5 % normal goat serum for 30 min prior to the incubation with the primary antibody in a 1:30 dilution for 1 h at room temperature. A secondary biotinylated rabbit anti-rat antibody (E0468, DAKO, Glostrup, DK, dilution 1:100) was applied for 30 min at room temperature. Streptavidin-peroxidase-based detection was performed with the Dako REAL^TM^ Detection system Peroxidase/DAB+ kit (K5001, DAKO, Glostrup, DK). Serum and antibodies were diluted in Dako REAL^TM^ Antibody Diluent (S2022, DAKO, Glostrup, DK). Appropriate positive and negative controls were included in each reaction.

**Western Blot**

The specificity of the NOTCH2 antibody was confirmed by Western blotting using total protein from the gastric cancer cell line MKN28. T-Per buffer (Pierce, Rockford, USA) containing protease inhibitors (Roche Diagnostics, Mannheim, D) was used to lyse 1.5x10^6^ cells. 50 µg of protein was resolved by 10 % SDS-PAGE and blotted to a nitrocellulose membrane. The membrane was blocked with 5 % dry milk in TBST and incubated with the primary anti-NOTCH2 antibody (dilution 1:250) followed by an incubation with a HRP-conjugated goat anti-rat F(ab’)_2_ fragment (1:5,000 Amersham Bioscience, Freiburg, D). Signal detection was performed with ChemiGlow West chemiluminescence substrate and the FluorChem SP imaging system (Protein Simple, Santa Clara, USA).

**Analysis of public microarray data**

Publically available gene expression array data of gastric carcinomas [2] were analysed using BRB-Array Tools [3]. *POLR2A* and *IPO8* were included in the data set and were selected as reference genes. Normalisation to the reference genes was performed using the probes with the expression values exhibiting the minimal inter quartile range (IQR) and the least missing values. Probes for the analysis of the target genes (*CTNNB1*, *GSK3B*, *NOTCH2*) were selected based on the expression values with maximal IQR after normalisation and the least missing values. Only advanced gastric carcinomas (T3/4, n=58) were analysed for optimal comparability with our patient group. Optimal cut-off values of gene expression were determined for an association with patient survival by conditional inference tests for maximally selected statistics.

**Calculation of risk scores**

A risk score was calculated based on a multivariate Cox regression model essentially as described by Cho et al. [4]. This score was formed by summarising the products of the multiplication of the Cox regression coefficient of each gene in the model (Tables S4 and S6) by the corresponding normalised relative gene expression value (ΔΔC_t_) for each patient.

The formulas for the own and publically available data are as follows:

**Risk Score_own data_** = (-0.1913 x CTNNB1 value) + (-1.568 x GSK3B value) + (1.2036 x NOTCH2 value)

**Risk Score_public data_** = (-0.1496 x CTNNB1 value) + (-0.2009 x GSK3B value) + (0.1005 x NOTCH2 value)

Optimal cut-off values for dichotomisation into high and low risk groups were calculated by log-rank-statistics.

Supplementary References

1. Zagouras P, Stifani S, Blaumueller CM Carcangiu ML, [Artavanis-Tsakonas S](http://www.ncbi.nlm.nih.gov/pubmed?term="Artavanis-Tsakonas S"%5BAuthor%5D) (1995) Alterations in Notch signaling in neoplastic lesions of the human cervix. Proc Natl Acad Sci 92: 6414-6418.

2. Chen X, Leung SY, Yuen ST, Chu KM, Ji J, et al. (2003) Variation in gene expression patterns in human gastric cancers. Mol Biol Cell 14: 3208-3215.

3. Zhao Y, Simon R (2008) BRB-ArrayTools Data Archive for human cancer gene expression: a unique and efficient data sharing resource. Cancer Inform 6: 9-15.

4. Cho JY, LIM JY, Cheong JH, Park YY, Yoon SL et al. (2011) Gene expression signature-based prognostic risk score in gastric cancer. Clin Cancer Res 17: 1850-1857.
